# Systems view on spatial planning and perception based on invariants in agent-environment dynamics

**DOI:** 10.3389/fnins.2014.00439

**Published:** 2015-01-13

**Authors:** Bérénice Mettler, Zhaodan Kong, Bin Li, Jonathan Andersh

**Affiliations:** ^1^Interactive Guidance and Control Lab, Department of Aerospace Engineering and Mechanics, University of MinnesotaMinneapolis, MN, USA; ^2^Department of Mechanical Engineering, Boston UniversityBoston, MA, USA; ^3^Department of Computer Science and Engineering, University of MinnesotaMinneapolis, MN, USA

**Keywords:** guidance, perception, decision making, visual attention, dynamics

## Abstract

Modeling agile and versatile spatial behavior remains a challenging task, due to the intricate coupling of planning, control, and perceptual processes. Previous results have shown that humans plan and organize their guidance behavior by exploiting patterns in the interactions between agent or organism and the environment. These patterns, described under the concept of Interaction Patterns (IPs), capture invariants arising from equivalences and symmetries in the interaction with the environment, as well as effects arising from intrinsic properties of human control and guidance processes, such as perceptual guidance mechanisms. The paper takes a *systems' perspective*, considering the IP as a unit of organization, and builds on its properties to present a hierarchical model that delineates the planning, control, and perceptual processes and their integration. The model's planning process is further elaborated by showing that the IP can be abstracted, using spatial time-to-go functions. The perceptual processes are elaborated from the hierarchical model. The paper provides experimental support for the model's ability to predict the spatial organization of behavior and the perceptual processes.

## 1. Introduction

Spatial control tasks require comprehensive planning and control strategies to deal with the uncertainties and disturbances in the immediate surroundings, while accounting for and anticipating various known and unknown elements in the larger task environment. Examples of such tasks are as varied as piloting in confined environments or performing surgery. Most of these tasks require similar basic skill components. These include the ability to control a dynamical system (the limb, body, surgical instrument, or aircraft) to achieve useful movements or maneuvers, as well as planning how these maneuvers should be deployed in relationship to task elements. While engaging in these activities, a pilot or operator also has to mitigate effects of disturbances, uncertainties in the task conditions, uncertainties in the system's dynamics, as well as contingencies affecting the larger task elements and goal (Mettler et al., 2010; Mettler, 2011).

Consequently, human versatility and agility in guidance must rely on harmonious integration of sensory and control functions with high-level cognitive planning processes. Taken as a whole, this system involves both continuous and discrete processes, as well as deterministic and probabilistic ones. Besides this *system complexity*, there is also a *behavioral complexity*, which arises from the high-dimensional, stochastic, and non-linear dynamics. In transportation, these dynamics combine the interactions between vehicles or agents with the task environments. Spatial performance under these conditions is an emergent phenomenon. This makes such systems difficult to understand and challenging, if not impossible, to model via traditional modeling techniques, such as those used in robotics or control engineering. Therefore, any theory of human guidance behavior must first address the *system* and *behavioral complexities*.

The fundamental questions that motivate this research involve establishing a correct, high-level description of behavior. For example, can behavior be described in terms of subgoal sequence? What are the principles that govern high-level descriptions such as subgoals? How can these principles be used to generate plans? What are the perceptual functions, how do they relate to behavioral components, and how are they integrated with the control and planning functions?

To be effective, the control, perceptual, and planning processes must be structured enough to function in a systematic, organized fashion, while allowing for enough flexibility for continuous and efficient adaptation. When controlling agile vehicles or movements, human operators must learn behaviors that can be efficiently described or codified and are simple to implement. It is reasonable to assume that in order to mitigate complexity, the brain relies on spatial and temporal structures inherent to guidance behavior. It is also reasonable to assume that these structural forms are closely related to physical and biological constraints at play in the larger system.

Elucidating principles that underlie the organization of behavior is an old problem in cognitive sciences (Miller et al., 1960). The concept of *motor equivalence* has long remained an influential idea (Hebb, 1949), however, it is often used without clear principle (Munhall, 1986). The problem gained some clarity with the motor control perspective. Behavioral complexity has been described as the “degree of freedom” problem (Bernstein, 1967) by Bernstein, who proposed that joint and muscle behavior are organized in a low-dimensional subspace, which he called *muscle synergies*. Consequently researchers have been interested in decomposing behavior into elemental components, which could help elucidate the principles of macroscopic organization of behavior, as well as the organization of the system itself.

Similar issues have come up in early motion programming and trajectory optimization (Bryson, 1996). Bellman's “curse of dimensionality” (Bellman and Dreyfus, 1962) describes the complexity arising from brute force discretization of motion, as it is typically found in numerical trajectory optimization (Betts, 2001). Therefore, in the engineering field, researchers have been interested in finding motion primitives that can efficiently describe motion (see e.g., Frazzoli et al., 2002). However, both behavioral sciences and engineering generally tend to focus on the individual control, sensing, or planning processes, without considering the larger system interactions, in particular the larger agent-environment coupling.

In summary, when humans operate in natural environments, such as piloting in complex terrains or performing surgery, they have to learn the patterns of interaction between the environment and motion, as well as learning how to extract useful visual cues. *Interaction Patterns* (IPs), which are structural features emerging from the dynamical interactions in the agent-environment system, have recently been proposed as a way to formalize these concepts (Kong and Mettler, 2013). In addition, humans need to learn to exploit the structure these patterns confer, in order to organize behavior in ways that mitigate the various sources of complexity. Therefore, a key modeling task is to delineate between these levels and detail the organization of the system. Invariants in this larger system are expected to play a central role in shaping the architecture responsible for integrating controls, perception, and planning processes.

The paper builds on the concept of IPs to give a comprehensive *systems' perspective* on the integration of the planning, control, and perceptual processes. The rest of the paper is organized as follows: Section 2 provides a brief overview of related work in the fields of cognitive and neurosciences, robotics, and machine learning. Section 3 describes and illustrates the concept of IPs and introduces the experiment used throughout this paper. In Section 4 the properties of IPs are used to delineate a hierarchical model that unifies planning, perceptual guidance, and control, while detailing the different information elements and control functions. Section 5 describes how the behavior associated with IPs can be abstracted and used to realize efficient motion planning. Section 6 then uses the functional understanding gained from the model to detail the perceptual mechanisms. Supporting evidence is provided by investigating patterns found in experimental gaze tracking data. Finally, Sections 7 provides a discussion of the relevance of the key results to the study of human spatial behavior and 8 provides general conclusions.

## 2. Background

The present research is primarily motivated by the persisting gap between the capabilities of skilled human operators, or pilots, and automation. This section provides background from various relevant disciplines.

### 2.1. Human control engineering

The theory of human control behavior goes back to the 1930–40s, when Tustin characterized the human as a feedback servo element (Tustin, 1947). The feedback theory was soon applied to modeling pilot/vehicle system. The most significant result was McRuer's crossover model, which describes human feedback control behavior as a linear transfer function (McRuer and Krendel, 1974). McRuer showed that for a large variety of controlled systems, the plant under the effect of the human control results in the same general loop transfer function *L*(*s*) = *K*/*s* (McRuer, 1980). These models focus on tracking and pursuit tasks, in which subjects track a given visual stimuli.

More comprehensive models have been proposed, such as the multi-loop model. That model describes pilots' manual rotorcraft control in approaches to hover (Heffley, 1979). The feedback loops are organized hierarchically starting with the low-level attitude stabilization to tracking and goal-directed maneuvering. However, these models still assume knowledge of a goal state and therefore don't provide a complete theory for spatial control behavior.

One of the most studied domains of human control behavior encompassing the larger control hierarchy is driving. In driving, a significant part of the behavior takes place within a well-defined traffic and driving rule structure. This structure allows researchers to formalize the problem in terms of explicit quantities (traffic signs, driving lanes, etc) and helps to model the control, perceptual, and planning processes. In Michon (1985), the authors exploit the structure in the environment and traffic system to model the human driver, delineating cognitive functions from a human problem-solving perspective. In another example Macadam (2003), authors have been able to incorporate biophysical limitations related to visual, control delay, and information processing. More recent research has emphasized data-driven approaches. For example, in Terada et al. (2010) a hierarchical identification scheme is used to model driver behavior without predefined behavioral modes.

### 2.2. Formal motion models and abstractions

In engineering, various discrete abstractions have been proposed to enable more efficient robot motion planning and control. Brockett's motion description language (MDL) (Brockett, 1990, 1993) represents the classic formal language for robot programming. It combines discrete modes with behaviors described by dynamic models. With these types of models, the emphasis is mainly on the mathematical and computational formulation. Models emphasizing the dynamics, for example, for application of agile vehicle guidance, include the motion primitive automaton (MPA) (Frazzoli et al., 2002). The motion primitives in this type of MPA correspond to a quantization of the vehicle dynamics, i.e., equilibrium trajectories and maneuvers. The interaction with the environment is solved during the generation of the trajectories. There are also methods that are derived from discretizations of the environment (Belta et al., 2005) and typically rely on nested control policies. As these examples illustrate, typical forms of abstractions focus either on the agent's dynamics or the environment and therefore don't capture the important agent-environment interactions.

### 2.3. Perception and attention in guidance

The school of ecological psychology was the first to emphasize agent-environment coupling, going back to Gibson, who highlighted the mutuality between perception and action (Gibson, 1979). The idea was that both response to action and perception of the environment obey lawful principles and that their mutual or reciprocal effect is exploited to achieve effective perceptual control mechanisms. Researchers have explained so-called *lawful* relationships between movement and perception and between action and movement (Turvey and Carello, 1986).

The most broadly established theory of perception in the context of guidance is tau theory (Lee, 1998). This theory, which originates from Gibson's ecological psychology, provides a direct link between perceived quantities and control action. It has been validated in various animal behaviors, as well as more recently in pilots (Padfield et al., 2003, 2012). The strength of this mechanism is its simplicity, which enables real-time implementation. A general shortcoming of perceptual theories, however, is that they do not provide an understanding of the larger planning and reasoning processes. In the following experiments, perceptual guidance mechanisms can viewed as constraints in spatial behavior, associated to human perceptual mechanisms, and manifest as invariants in behavioral patterns.

In humans, attention models have primarily focused on still images (see for e.g., the benchmark eye movement datasets Borji and Itti, 2013). Visual attention in the context of guidance behavior represents another research direction. Significant progress has been made in understanding insect sensory guidance systems (Srinivasan et al., 2006; van Breugel and Dickinson, 2012), however, these creatures occupy vastly different environments than humans and are confronted with more restricted planning and adaptation challenges.

The author's investigation of the lower-level coupling between control action and visual gaze, in remote control tasks using miniature rotorcraft, has been described in Andersh et al. (2014). The results demonstrate the role of gaze in providing both measurement updates for estimating the rotorcraft state necessary for accurate tracking and anticipatory information for goal interception. These results are consistent with the perceptual process described in Section 6.

### 2.4. Embedded dynamics' view

Guidance involves a range of dynamical interactions, starting with those inherent to the vehicle or body and then extending into the dynamics of the entire human-machine or agent-environment interactions. Principles from dynamics and control are in operation across the entire system. Inspired in part by the ecological view, researchers have formalized behavior as the embedded, closed-loop, agent-environment interaction (Warren and Fajen, 2004). Taking a larger systems view, information is extracted and fed back at multiple levels.

Following the ecological psychology movement, researchers have grown interested in a more formal dynamics and control-based theory of perception and action. A notable example is Warren's control theory that integrates the dynamics of action and perception (Warren and Fajen, 2004; Warren, 2006). Warren's model assumes that the necessary information for the control action regulation is extracted from the environment, with no need for a fully developed environment model. Four main ideas are integrated: (i) the agent is embedded in the environment, (ii) control is determined with information about the agent-environment state, (iii) control actions are specific to the current task, and (iv) behaviors result from agent-environment interactions.

Applications of this model have mostly focused on simple tasks such as balancing an object, bouncing a ball on a racket, or walking. A well-known example is the catching of a fly ball by a baseball outfielder (McBeath et al., 1995). The analysis of behavior as an embedded dynamical system, along with its associated closed-loop model (Equation 1), also leaves many details to be elucidated. These include information extraction laws and the type of control structures and their associated input, output, and state variables. Due to the *systems complexity*, these are not as simple to identify in an embedded dynamical model as they are in traditional feedback control systems.

### 2.5. Invariants in motor and perceptual behavior

Given the variability and often apparent lack of structure in human behavior, it is often useful to determine which aspects of behavior are *invariant* and which are *variable*. An early concept related to these questions was referred to as *motor equivalence*. Its original meaning was that the same outcome can be achieved through different motor actions (Hebb, 1949). The concept is also associated with Bernstein's *degree of freedom problem*. Bernstein suggested that muscles are organized in so-called *synergies*, i.e., patterns of activations that provide low-dimensional descriptions. Modeling efforts have primarily been successful at describing simple movement characteristics in motor control, such as speed/accuracy properties (Fitts, 1954; Bullock and Grossberg, 1988) and invariant features in the movement kinematics (Flash and Hogan, 1985).

The concept of motor equivalences can be extended to the problem of control of spatial behavior in general, such as the flight trajectories, as well as the control of vehicles and other mechanisms. In analogy to “muscle synergies” in motor behavior, the goal is to identify “guidance synergies” that mitigate complexity and provide a structure for adaptation. The mappings of perceptual invariants to movement kinetic invariants, as identified by Kugler and Turvey (1987), represent an example of such low-dimensional subspaces in the agent-environment dynamics. However, these synergies have to be investigated from the perspective of the larger behavior organization and planning. Therefore, investigating spatial and temporal regularities and other characteristic structures can unveil more details about the various system processes and their organization.

### 2.6. Neuro-cognition of interactive behavior

The paper's questions of parameterization of high-level behavior specifications and planning mechanisms are closely related to classic as well as recent perspectives in cognitive neuroscience.

A common principle of human information processing is that of chunking. Identifying subgoals, and more generally, decomposing tasks into subtasks or stages, constitute a common principle in planning across various domains (Newell, 1994). The existing concepts of subgoals, however, do not explain how these are actually determined. Subgoals are also commonly used in robotic flight, where many navigation algorithms rely on the insertion of way points. These subgoals, however, are typically determined from heuristics, such as visibility graphs (de Berg et al., 2008).

Hierarchical principles still appear preeminently in the neuro-cognitive literature, such as the hierarchical process of the perception-action cycle (Badre, 2008) or the cerebral mechanisms of reaching motion (Kalaska and Crammond, 1992). While many brain processes and sub-systems are well-defined within this framework, there are also many interactions between these processes. One example is the interaction between visual processing and goal specification and planning, for which “massive reentrant circuits” call for more definition (Mountcastle, 1978). Cisek and Kalaska (2010) give a comprehensive description of the neuro-cognition of interactive behaviors and challenge the traditional serial information processing model. The authors advocate investigating the process of attention and action selection/specification from interactions in brain activities.

### 2.7. Machine learning

The primary objective of machine learning has generally been to replicate human behavior. The multiple processes and biological constraints of guidance, however, make it challenging to formulate the problem as a reinforcement learning process. So far, results have been reported about specific aspects, such as maneuvering (Abbeel et al., 2007). One promising direction has been to exploit hierarchical characteristics of behavioral trajectories (Barto and Mahadevan, 2003). Similarly, in the field of deep learning, researchers are developing methods to extract more dynamic and useful information by automatically learning feature hierarchies (Hinton et al., 2006; Bengio, 2009), which directly relate to the concepts of equivalences, and if applied to behavioral data, could provide valuable insights.

## 3. Patterns in agent-environment dynamics

This section provides a formal definition of patterns in guidance behavior which will be used as building blocks for the systems level analysis. The section also defines a representative example and describes the experimental data used throughout the paper to illustrate various aspects of the material. The section concludes with remarks on the significance of these results from a cognitive standpoint.

### 3.1. Representative human spatial control task

A typical spatial control problem is piloting a helicopter in a challenging environment, such as the problem a medevac pilot is confronted to when operating in an urban or other confined terrain^1^. The helicopter dynamics are given by the equations of motion *ẋ* = *f*(*x*, *u*), where *x* ∈ 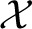 ⊆ ℝ^*n*^ is the state of the agent and *u* is the control action. 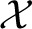 is the *state space*, which in the interest of conciseness is assumed to be finite or countable. The state of an agent is a minimal set of variables that can determine the behavior of the agent. In this example, the state *x* of the pilot-helicopter system can be the position, orientation, linear, and angular velocity of the helicopter.

A typical engineering approach would solve this problem as a minimum-time trajectory optimization problem Bryson and Ho (1975). In the medevac example, *x*_0_ could be the patient pickup location and *x*_*g*_ the location of the hospital. The minimum-time control u^*^(*x*_0_, *x*_*g*_) with these two boundary conditions minimizes *T*_*x*_*g*__(*x*_0_), the time it takes from *x*_0_ to *x*_*g*_. This example represents a typical two-point boundary value problem found in trajectory optimization.

In this example, T^*^_x_*g*__(*x*_0_) is the pilot's minimum flight time to the hospital and u^*^_x_*g*__(*x*_0_) is a description of the state and action sequence, e.g., where to turn, how aggressively, at what speed, etc. Similarly, given a goal state *x*_*g*_, an optimal time T^*^_x_*g*__(*x*) can be defined for any state *x* ∈ 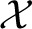. The set {T^*^_x_*g*__(*x*)|*x* ∈ 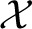} is called time-to-go (TTG) map Mettler et al. (2010). For the medevac example, 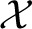 could represent the city's entire airspace and the associated TTG map gives the optimal flight time to reach the designated goal *x*_*g*_ from each location *x* in the city's airspace. Computing the TTG map can be computationally heavy and is most likely not how human pilots solve the guidance problem.

#### 3.1.1. Definition of guidance behavior

*Guidance behavior* is defined as the collection of all closed-loop state trajectories resulting from the agent's interaction with the environment (Kong and Mettler, 2011, 2013). These state trajectories can be written as follows:

(1) $$\dot{x}=f(x,k(x,h(g(x))))$$

The above definition of guidance behavior is inspired by the ecological perspective on perception and action (Turvey and Carello, 1986) and more specifically Warren effort to formalize the relationship between action and perception (Warren, 2006). It captures the following key processes: *g*(.) describes how the agent affects the environment state, *h*(.) describes how the agent abstracts information from the environment, and *k*(., .) is the control policy used by the agent. Notice that the output of function *g*(.), which captures the physical interaction between the agent and the environment, is different from the output of function *h*(.), which captures how the agent perceives the interaction. We call the output of function *g*(.) the environment state, i.e., *e*(*t*) = *g*(*x*(*t*)), where *e*(*t*) ∈ 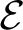, with 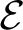 as the *environment state space*.

Consider the pilot in the example, who is controlling the helicopter to the hospital landing pad. *e* or *g*(*x*) is some environment state that is affected by the motion of the helicopter, for example the distance between the helicopter and the landing pad. *h*(*g*(*x*)) is the pilot's perception of the helicopter-environment interaction, which can be optical flow or tau (Lee, 1998). *u* = *k*(*x*, *h*(*g*(*x*))) is then the pilot's control, based on his or her perception of the environment and his or her own motion. Finally *f*(*x*, *k*(*x*, *h*(*g*(*x*)))) are the dynamics of the helicopter, which determine how the state evolves, given the current state *x* and a control input *u*.

#### 3.1.2. Mechanisms for mitigating complexity

Complexity (Bellman's “curse of dimensionality” and Bernstein's “Degree of Freedom problem”) makes computing solutions to the planning problem intractable for rapid decision making and adaptation. Complexity can be mitigated by exploiting structure in the problem space, as well as process constraints, e.g., accounting for how humans guide and control motion, how the visual system processes environment cues, etc. Important classes of structural elements in any problem are similarities or equivalence classes. For example, the way a pilot or driver negotiates a particular obstacle is similar to how he negotiates other obstacles. This similarity pattern, combined with properties of symmetry group, e.g., the invariance of this obstacle avoidance strategy under rotation and translation, makes it possible to decompose the problem space into a sequence of obstacle avoidance maneuvers.

### 3.2. Equivalences and concept of subgoals in guidance behavior

To formalize the notions of “invariances” and “equivalences” in spatial control, as well as the associated notion of “subgoal,” it is necessary to define two forms of equivalences as they relate to guidance behavior. The description uses formalisms from computational mechanics (Shalizi and Crutchfield, 2001). Further details are available in Kong and Mettler (2013).

Following the definition of guidance behavior an *extended state space* 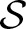 is defined as the product space of the previously introduced state space 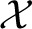 and environment state space 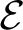, i.e., 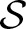: = 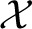 × 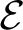. The following explanation uses the notations used in computational mechanics. $\overset{\leftarrow }{.}$ and $\vec{.}$ represent the temporal direction of a state sequence. The subscript denotes the time index. For instance, $\overset{\leftarrow }{s_{i}}$: = … *s*_*i* − 2_ *s*_*i* − 1_ *s*_*i*_ and $\vec{s_{i}}$: = *s*_*i*_ *s*_*i* + 1_ *s*_*i* + 2_ … with *s* ∈ 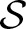. Finally 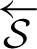 and 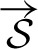 are the collections of all state sequences in the form of $\overset{\leftarrow }{s_{i}}$ and $\vec{s_{i}}$, respectively. Notice that in reality both $\overset{\leftarrow }{s_{i}}$ and $\vec{s_{i}}$ are of finite length.

The *first equivalence relationship* ~_*s*_ is defined using the concept of causal state (Shalizi and Crutchfield, 2001):

(2) $$\overset{\leftarrow }{s_{i}}\sim_{s}\overset{\leftarrow }{s_{j}}\Leftrightarrow \left(\vec{s}|\overset{\leftarrow }{s_{i}}\right)=\left(\vec{s}|\overset{\leftarrow }{s_{j}}\right)$$

for all $\vec{s}$ = *s*_0_ *s*_1_ …. This equation states that if starting from the state *s*_0_, the two trajectories $\overset{\leftarrow }{s_{i}}$ and $\overset{\leftarrow }{s_{j}}$ follow the same trajectory $\vec{s}$, then these two trajectories are equivalent in the sense of ~_*s*_, i.e., $\overset{\leftarrow }{s_{i}}$ ~_*s*_ $\overset{\leftarrow }{s_{j}}$. Furthermore, the state *s*_0_ is called the *subgoal* associated with trajectories $\overset{\leftarrow }{s_{i}}$ and $\overset{\leftarrow }{s_{j}}$.

The *second equivalence relationship* ~_*g*_ is based on the symmetry group associated with rigid-body dynamics. In the present case, the symmetry group is in the form of a finite-dimensional Lie group *M*. The action (read: transformation) of this Lie group *M* on 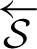 is Ψ: *M* × 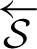 → 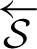. Two trajectories, $\overset{\leftarrow }{s_{i}}$ and $\overset{\leftarrow }{s_{j}}$, are equivalent in the sense of ~_*g*_, $\overset{\leftarrow }{s_{i}}$ ~_*g*_ $\overset{\leftarrow }{s_{j}}$, if there exists an element *m* ∈ *M* and control histories $\overset{\leftarrow }{u}$_*i*_ and $\overset{\leftarrow }{u}$_*j*_ such that:

(3) $$\left(\Psi \left(m,{\overset{\leftarrow }{s}}_{i}\right),{\overset{\leftarrow }{u}}_{i}\right)=\left({\overset{\leftarrow }{s}}_{j},{\overset{\leftarrow }{u}}_{j}\right).$$

Most vehicles are built around certain symmetry groups. For instance, the symmetry group of a helicopter is *SE*(2), which corresponds to a two-dimensional translation and a rotation with respect to the gravity direction. Such a symmetry group means that if a helicopter is flown with the same control at two different locations and in two different directions, the trajectories can be superimposed through a two-dimensional translation and a rotation about the vertical axis. The above definition similarly defines symmetry groups over the space 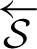.

### 3.3. Equivalence classes and interaction patterns

These two equivalence relationships, one defined based on symmetry groups (Equation 3) and the other one defined based on subgoals (Equation 2), formally define the *Interaction Patterns* (IPs) concept. The IPs represent the equivalence classes of guidance behavior 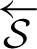, which can be understood as the quotient sets of 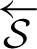 by ~_*s*_ and ~_*g*_. To be more specific, for a specific individual history $\overset{\leftarrow }{s}$ ∈ $\overset{\leftarrow }{S}$, an IP containing $\overset{\leftarrow }{s}$ can be defined as [[$\overset{\leftarrow }{s}$]] (Kong and Mettler, 2013), where

(4) $$\left[\left[\overset{\leftarrow }{s}\right]\right]=\left\{{\left[\overset{\leftarrow }{s}\right]}^{\prime }\in \left[\overset{\leftarrow }{s}\right]:{\left[\overset{\leftarrow }{s}\right]}^{\prime }\sim_{g}\left[\overset{\leftarrow }{s}\right]\right\}$$

and

(5) $$\left[\overset{\leftarrow }{s}\right]=\left\{{\overset{\leftarrow }{s}}^{\prime }\in \overset{\leftarrow }{S}:{\overset{\leftarrow }{s}}^{\prime }\sim_{s}\overset{\leftarrow }{s}\right\}.$$

We call [$\overset{\leftarrow }{s}$] a candidate IP. The reason is that in reality, a perfect superimposition of two candidate IPs, as defined by ~_*g*_, is impossible, due to various uncertainties and noises that are inherent in any human's sensori-motor system, as well as the vehicle under control.

An essential consequence of the IP definition is that it leads to a parsimonious representation of the extended state space 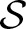. Since the dynamics in Equation 1 are deterministic, i.e., an initial condition *s*_0_ and the specific control law *k*(.) result in one unique trajectory. Therefore, it is possible to equate *s*_0_ with the trajectory $\vec{s_{0}}$. Thus, the following equivalence can be defined over states in 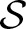, based on the IP definition (Equation 4):

(6) $$s_{i}\sim s_{j}\text{ if and only if }\left[\left[\vec{s_{i}}\right]\right]=\left[\left[\vec{s_{j}}\right]\right],$$

i.e., two states *s*_*i*_ and *s*_*j*_ are equivalent with respect to each other, if and only if the two trajectories $\vec{s_{i}}$ and $\vec{s_{j}}$ starting from these states belong to the same IP. Consequently, such an equivalent relationship results in an equivalence class set over 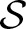. In the following, with a slight abuse of terminology, these equivalence classes will also be called IPs.

### 3.4. Experimental illustration of interaction patterns

**Figure 1:**
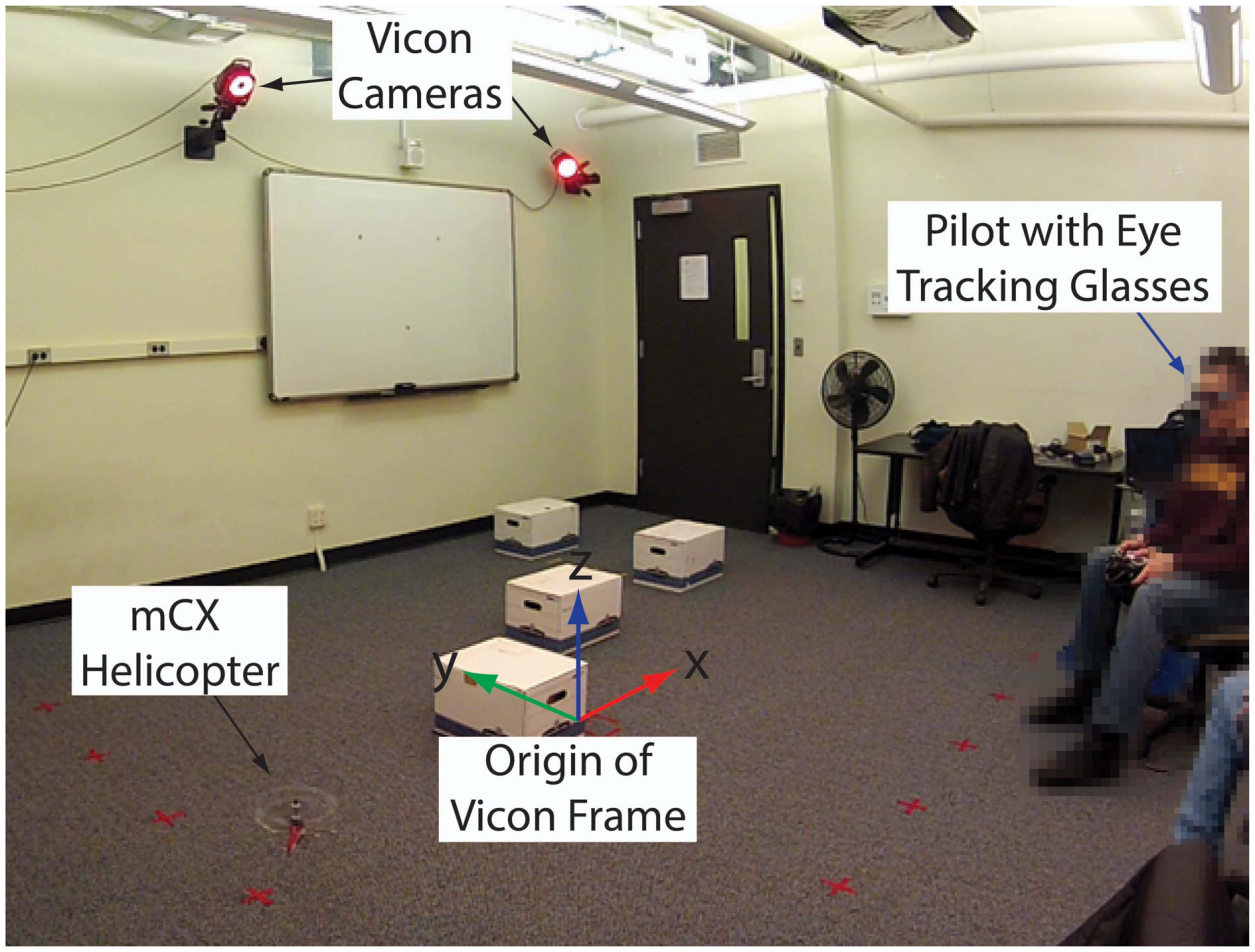
**Picture of the experimental facility**. Human guidance capabilities are investigated using experiments with miniature helicopters in an obstacle field. The helicopter motion is recorded using a Vicon motion tracking system and the operator gaze is recorded using SensoMotoric eye tracking glasses.

These concepts of equivalence classes have been applied to analyzing human guidance behavior. The guidance experiments were conducted at the Interactive Guidance and Control Lab (IGCL) shown in Figure 1 using miniature helicopters. The guidance experiments consist of precision goal interception tasks in an obstacle field. Entire trajectory ensembles are collected to cover the entire task space. This is achieved by using a grid of starting points and then running repeated trials from each starting point. The helicopter state at the goal must satisfy a position and terminal velocity tolerances (Mettler and Kong, 2013).

These aircraft enable broad expression for a subject's spatial guidance skills and hence are ideal to investigating the agent-environment interactions. The experiments are treated as a planar guidance problem where the agent's state is *x* = [*s*_*x*_, *s*_*y*_, *v*, ψ]′, where *s*_*x*_ and *s*_*y*_ are the helicopter positions and *v* and ψ are its speed and course angle, respectively. The measurements are obtained from a Vicon motion tracking system.

**Figure 2:**
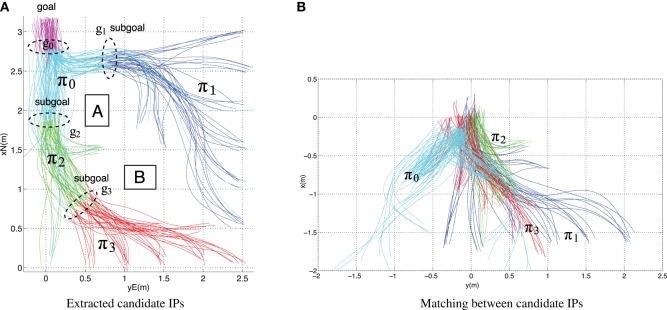
**(A)** Trajectories highlighted based on corresponding candidate IPs (π_0_, …, π_3_). The candidate IP determines the partitioning of behavior and associated subgoal areas. **(B)** Overlay between extracted candidate IPs obtained through rotation and translation of π_0_, …, π_3_. The matching indicates the degree of equivalence in behavior patterns, i.e., for this experiment, the candidate IP π_0_, …, π_3_ can be regarded as belonging to the same IP.

Figure 2A shows trajectories collected from the guidance experiments and highlights candidate IPs extracted, based on the equivalence classes (see Kong and Mettler, 2013 for details).

Together, the IPs fully describe the guidance behavior.

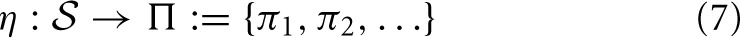



The set of IPs can be combined under the concept of guidance primitives, with Π describing the guidance primitives library (Kong and Mettler, 2013).

Figure 2B shows the overlaid IP candidate obtained by applying symmetry transformation (rigid-body translation and rotation). The matching between the candidate IPs underscores the similarities in strategies, i.e., human operators exploit the equivalence in the problem to organize the guidance behavior. In other words, the similarity among the candidate IPs π_0_, …, π_3_ (formally π_0_ ~_*g*_ π_1_ ~_*g*_ π_2_ ~_*g*_ π_3_) means that for this experiment they can be considered as belonging to the same IP.

### 3.5. Significance for cognition and planning

The IPs make the operator's organization visible. In particular, the IPs make it possible to decompose the larger guidance task into a sequence of sub-tasks, each characterized by an IP and its associated subgoal (Mettler and Kong, 2012; Kong and Mettler, 2013). Therefore, to make an analogy with muscle synergies, the hypothesis is that the brain organizes and adapts patterns of trajectories, instead of the low-level dynamics.

In regard to chunking, the IPs describe how otherwise unrelated trajectories are aggregated, based on the equivalence principle. The principle of equivalence elaborated here suggests how the process of chunking of behavior may serve to improve information processing. The significance of IPs from a cognitive standpoint is that they make it possible to use only partial information for planning, i.e., they abstract the details associated with sensing and control to achieve more efficient planning and organization of behavior.

With regard to subgoals, the IP shows that the subgoals are an emergent phenomenon that follow from operators exploiting invariants in the agent-environment dynamics. This emergent phenomenon involves mutual bottom-up and top-down effects. The IPs result from the reciprocal effects between the perceptual and control constraints responsible for the implementation that take place at the motor level and the association process responsible for formation of behavior units that take place at the cognitive level. This system is essentially motivated by the necessity of achieving efficient and adaptive plans with limited computational and storage resources.

In summary, the IPs provide general principles governing coordinated patterns of behavior in complex, unstructured environments, and furthermore, suggest details of the hierarchical model associated with guidance behavior.

## 4. Systems integration and hierarchical model

Hierarchical structures play a central role in many complex systems (Simon, 1962). IPs properties already establish their role as units of organization of behavior. In this section they are used as primary elements in the systems' integration, where they help delineate the hierarchy of control, perceptual, and planning functions.

### 4.1. Hierarchical multi-loop model

The general closed-loop model of behavior in Equation 1 is in reality a multi-loop system, composed of a planning mechanism, a perceptual guidance mechanism, and at the lowest level, a tracking/pursuit system used for motion implementation. This architecture unifies the planning model with two levels that have both been investigated and validated in human behavior: perceptual guidance (Lee, 1998; Padfield et al., 2012) and tracking or pursuit (McRuer and Jex, 1967).

**Figure 3:**
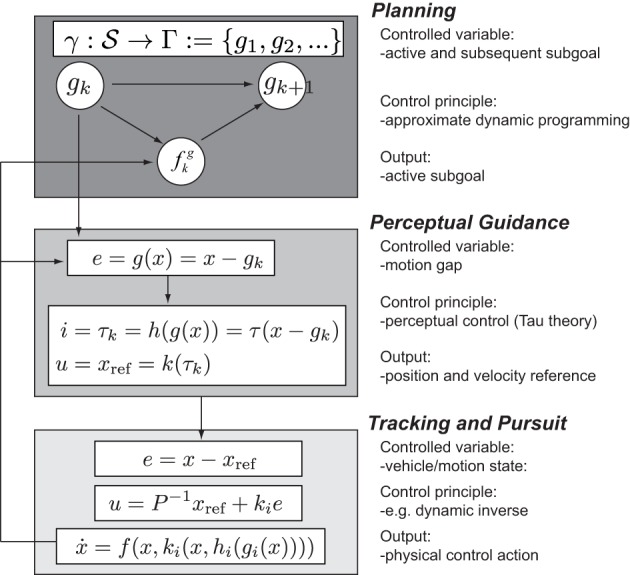
**Hierarchic multi-loop model of human guidance behavior**. The top level describes the planning level based on the decomposition of the task and environment in terms of the IPs. The plan is described as a subgoal sequence *g*_*k*_. The currently active subgoal defines the reference for the perceptual guidance. The latter extracts the current motion gap, which is used to determine a state reference trajectory *x*_ref_. At the lowest level, a tracking feedback system implements the desired motion for the vehicle.

The remaining modeling tasks are to detail each of these layers. The general idea is that each layer has its own respective environment models, information extraction laws, and control policies. The block diagram of the multi-loop architecture is illustrated in Figure 3. Complementary work investigating the lower levels of human guidance behavior is presented in Andersh et al. (2014).

#### 4.1.1. Planning: mapping of interaction pattern

Planning organizes the guidance behavior in terms of an arrangement of IPs. It represents the highest-level process in the model. By being used as a modeling language, IPs capture the complete control and perceptual interactions, suggesting that a mapping process exists which can transform current task configuration into a sequence of subgoals *g*_*k*_:

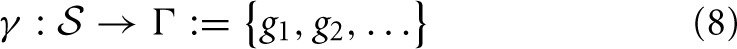



This mapping process can be seen as a type of pattern extraction mechanism, in which the basic principle is to associate the invariants in guidance behavior with the available modes of control and perceptual interactions (n.b. Gibson's affordances Gibson, 1979). Given the current system state *x* and partitioning of extended state-space 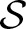 associated with the plan Γ, the output of this system is an active subgoal *g*_*k*_. The subgoal switching can be described by a state machine. One such planning mechanism is elaborated in Section 5.1.

#### 4.1.2. Perceptual guidance system

At mid-level, the active subgoal provides the environment representation *e* = *g*(*x*) = *x* − *g*_*k*_. Given the value of this gap, the agent implements an appropriate guidance policy to close the gap τ_*k*_. This task corresponds with an interception or coordination task, which can be modeled using a perceptual mechanism such as tau theory. The information-extraction mechanism determines the gap *i* = τ_*k*_ = *h*(*g*(*x*)) = τ(*x* − *g*_*k*_) associated with an active subgoal *g*_*k*_. This is similar to the mechanisms proposed as part of the perceptual guidance theory, but here the gap is based on the subgoal that is emerging from an IP-based decomposition. At this level, the control policy function is to generate a velocity reference that can be used by the tracking system.

#### 4.1.3. Tracking and pursuit system

At the bottom level is the control implementation. This system can be described by traditional feedback systems, such as determined from a dynamic inverse control design. Here it is specified by a feedback control gain *k*_*i*_ and the equilibrium state associated with the subgoal *g*_*k*_. The environment of the tracking system involves the speed and position perturbation associated with the reference and vehicle state. The information extraction consists of an error cue transformed into a desired acceleration. Finally, the control action from the feedback system is responsible for implementing the desired acceleration needed to close the motion gap.

## 5. Abstraction of interaction patterns and planning

This section illustrates how the IP can be used to plan guidance behavior efficiently. The general idea is that given a goal state in an environment with obstacles, the overall guidance behavior can be planned as a sequence of subgoals. These subgoals are determined by using partial information associated with the IP.

### 5.1. Planning problem and generation algorithm

Returning to the example in Section 3, the concept of IP can be used to simplify the TTG map generation and the guidance process given in Equation 8. First, instead of using the entire dynamic envelope of the piloted helicopter, the IP constrain the dynamics to the particular form of behavior associated with the agent environment interactions. The behavior in each IP can be abstracted by a TTG function. The global TTG map can then be obtained by using these TTG functions. Similar as with the muscle synergies, the IP provides a low-dimensional subspace for the pilot's organization of guidance behavior. Second, the construction of a TTG map, given a particular environment and IP set, can be decomposed into a sequence of sub-tasks, where each corresponds to the realization of an IP.

In the medevac example, IPs correspond to the set of maneuvers that the pilot performs when negotiating the various task and environment elements, e.g., circling around a building corner or bopping-up above a ground obstacle. The overall medevac mission can be achieved by a concatenation of these IPs and the typical“unconstrained” trajectories and maneuvers such as, straight and level flight and turning maneuver, respectively. These IPs are specific to the particular pilot's skill level.

Building on these ideas, an IP-based planning algorithm for predicting human guidance behavior can be conceived as follows. First, assume that a particular human's IP set is fixed and learned from his or her former experiences. Second, given a task with obstacles and a goal, the organization of IPs can be predicted by a wavefront algorithm (see Kong and Mettler, 2014 for details). The wavefronts describe how the TTG function propagates through an environment, interacts with obstacles, and how new wavefronts are generated through these interactions.

As a wavefront starts from the goal, the TTG level lines propagate according to the behavior associated to the IP (see e.g., the learned TTG in Figure 4A). As the wavefront collides with the vertex of an obstacle, a subgoal is created and a new wavefront starts from this subgoal. The multiple wavefronts interact. Colliding wavefronts form a ridge-like structure in the TTG map. This ridge defines locations where the trajectories can bifurcate, yet arrive at the goal in the same amount of time. The subgoals and ridges describe the organization associated with the IP.

**Figure 4:**
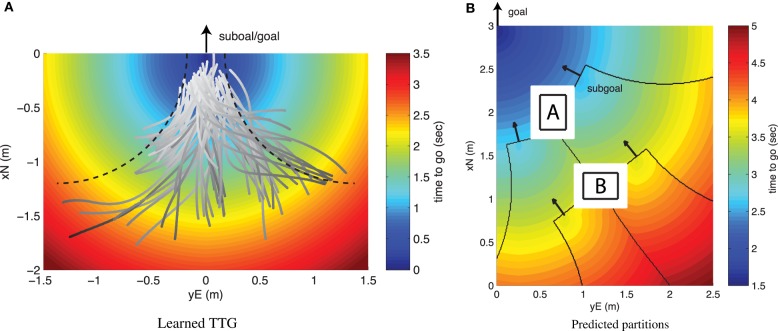
**(A)** TTG function learned from candidate IPs extracted from task in Figure 2A. The dashed line shows the boundary of the IP, assuming constrained normal acceleration and uniform motion. **(B)** Predicted organization of IPs based on the wavefront algorithm and the learned TTG.

### 5.2. Experimental evaluation of the subgoal prediction

Figure 4 illustrates the prediction of the guidance behavior, given in terms of sequence of subgoals, for the guidance experiment using the IP-based wavefront algorithm with TTG functions learned from IP.

The TTG for the IP is learned from the data collected from the trajectory segments associated with the IP extracted in Figure 2A. In this example, a single IP is sufficient to capture the pilot's skills (Kong and Mettler, 2013). A forward method with cross-validation was used to select the features needed to accurately learn the TTG map from the experimental data (Kong and Mettler, 2014). Figure 4A shows the IP's trajectory segment and learned TTG function.

The comparison between the extracted subgoals and partition shown in Figure 2A and the predicted subgoals and partition shown in Figure 4B, indicates that abstracting behavior using TTG functions and using these functions as part of a wavefront algorithms provides a relatively accurate and very efficient planning mechanism. This approach therefore provides a practical computational implementation incorporating necessary elements and information needed for planning.

### 5.3. Planning results discussion

The present experiment is based on stationary task elements. A single IP is sufficient to describe the overall performance. This is due to the relatively constant speed, hence subgoals have essentially the same terminal state. In more challenging tasks, the behavior may overlap with different subgoal values. More extensive investigations are needed, however, the same general approach and algorithm are expected to remain valid.

Human planning mechanisms, however, may be quite different. For example, they may not implement wavefront algorithms to determine the subgoal sequence. The main points are as follows. First, the IP is a finite-state representation similar to motion primitives that enable abstraction of the combined effects of human sensory, control, and perceptual capabilities. Compared with classic trajectory optimization, instead of learning the behavior over the entire space 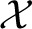, the IP make it possible to process the environment elements within a lower dimensional subspace, which results in a sequence of subgoals. Second, the abstraction of behavior in terms of IPs suggests structured mechanisms where behavior is adapted at the level of subgoals and within the IP. These mechanisms are primarily driven by perceptual information and are described next.

## 6. Experimental investigation of perceptual hierarchy

Planning, guidance, and control of behavior must be coupled efficiently with the perceptual processes used to extract necessary information. Perceptual guidance theory already provides precise understanding of this coupling for goal-directed guidance. When it comes to the larger system, however, we expect perception to be involved, both at the global situational awareness level, as well as the lower control implementation level.

The extracted IPs capture the overall agent-environment interactions, and are the by-product of the entire decision-making hierarchy, from perception, control, and guidance to higher-level planning. Investigation of visual attention provides the opportunity to understand these processes and their integration, i.e., to determine what information is used and exchanged at the different levels and how these different components of information are acquired.

**Figure 5:**
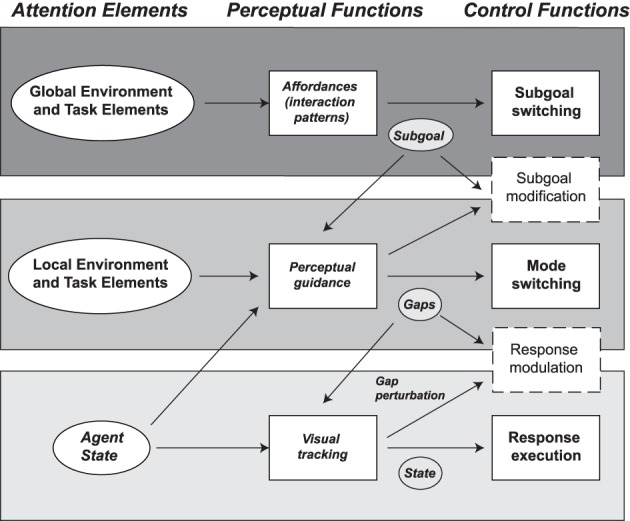
**Attentional model derived from the hierarchic model delineating the roles of the perceptual system ranging from the low-level visual tracking to the classic perceptual guidance and the higher-level perception**. The subgoal, motion gaps, and state (shown in shaded ovals) describe the information element that mediates between the perceptual and control functions.

Figure 5 shows an overview of the hierarchy of perceptual functions in guidance with the corresponding control functions. The following section describes the derivation of this perceptual model from the hierarchical model and provides preliminary experimental validation.

### 6.1. Visual attention in guidance behavior

Visual attention is the fundamental process associated with visual perception of the environment. Gaze is closely associated with attention and has several functions, including compensating for body movement, acquiring location data of a specific target, and providing information for visuo-motor control. Therefore, when processing gaze, it's important to distinguish these functions.

The fact that visual attention cannot be divided, combined with the typically dynamic and uncertain task elements, make attention allocation a fundamental aspect of human guidance behavior. While it has been well-documented that visual attention is driven by a combination of *top-down* and *bottom-up* perceptual processes, few investigations have elucidated their integration (Navalpakkam and Itti, 2006).

Existing literature indicates that gaze activity is proactive, typically seeking out information required to complete a task or action in the seconds preceding it Land et al. (1999); Hayhoe and Ballard (2005). Navigation tasks, like most planning problems, are a form of dynamic program (DP) (Bertsekas, 1995), i.e., predictions of the future actions, ideally all the way to the goal, are needed to determine current actions.

### 6.2. Attentional model and expected visual attention patterns

The model in Figure 5 provides a systematic description of the coupling between perceptual and control processes. The model describes the *Attention Elements*, *Perceptual Functions*, and *Control Functions* for the three hierarchic levels. The *Control Functions* are used at each level to determine necessary information mediating their operation. In turn, this information is used to define the *Perceptual Functions* and *Attention Elements*.

Starting from the top, the planning level's *Control Function* involves determining the current active subgoal *g*_*k*_ from the subsequent subgoals, all the way to the goal, i.e., *g*_*k* + 1_, … *g*_*goal*_. Since humans have limited working memory and attention, they are constrained in their ability to attend to many stages in the future and elements. The planning level's *Attention Elements* are the global environment elements and task elements. The model suggests that the *Perceptual Functions* process the environment and task elements to extract “IP affordances” associated with the IPs. At the guidance level, perceptual guidance theory is formulated in terms of the motion gap, which represents the information element. In related research (Andersh et al., 2014), it was found that the operator performs anticipatory saccades to determine the motion gap to targets. Regarding the control level, guidance also requires relatively continuous attention to the vehicle and immediate environment, in order to maintain control and modulate the response to changes in the local task or environment elements. Finally, for each of the three levels, the model also defines the adaptation/modulation mechanisms (shown as dashed boxes in Figure 5). The adaptation mechanisms are properties of the model but will need to be further investigated.

**Figure 6:**
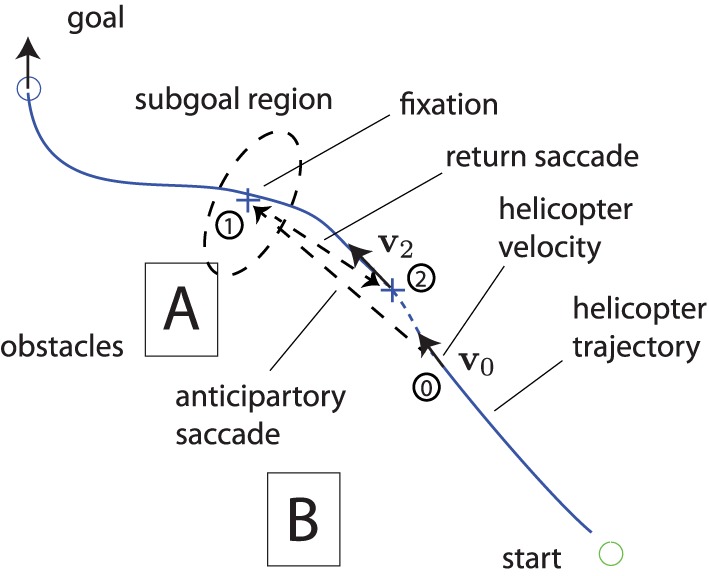
**Expected gaze pattern associated with the hierarchic multi-loop model**.

The primary *Control Functions* involve the determination and organization of IPs, motion guidance to the active subgoal, and the execution and modulation of the control response. The former requires perception of the larger environment and task elements. The latter focuses on the more immediate environment and task and how they relate to the active and subsequent subgoals. Regarding attention, the hypothesis is that IPs mediate top-down perception. Bottom-up perception is most likely mediated by saliency of the environment and task elements, including goal, obstacles, and vehicle. Based on these insights, the expected attention patterns should include anticipatory saccades directed at current and subsequent subgoals and smooth pursuit focusing on the helicopter. Figure 6 summarizes the gaze pattern expected in the guidance task.

### 6.3. Gaze tracking system

The Eye Tracking Glasses (ETG) from SensoMotoric Instruments (SMI, 2012) comprise binocular eye-tracking cameras, which automatically compensate for parallax and make gaze tracking accurate over a range of distances. A scene camera (1280 × 960 resolution at 24 frames per second) captures the operator's view. The ETG tracking covers a range of 80° horizontally and 60° vertically, with an accuracy of 0.5°. The scene camera field of view is 60° horizontally and 46° vertically.

Gaze-tracking data and video are streamed in real-time to a ROS-based data acquisition system (Quigley et al., 2009), where all the data (ETG gaze, ETG scene video, vehicle motion, pilot control inputs) are synchronized and stored.

### 6.4. Processing and registration of gaze tracking data

The ETG provide a gaze vector relative to the pose of the operator's head. A registration procedure is used to bring the operator's head, as well as helicopter, environment, and task elements, into the common reference frame shown in Figure 1. This data then allows investigation of the interactions between vehicle motion, operator control inputs, and visual attention.

The measurements provided by the ETG system include images captured by the forward-facing camera and gaze location coordinates, represented by pixel location w.r.t. to scene image. The gaze direction is converted to a vector in the ETG reference frame. Combining the gaze-tracking glasses with the Vicon motion-tracking system allows for mapping 2D gaze into 3D world and vice versa, taking tracked objects in the 3D world, and projecting their location onto the ETG's scene camera image plane.

The pose of the pilot's head is tracked by the Vicon system using reflective markers on the ETG. It's used in real-time to transform the gaze vector into the Vicon reference frame. Once expressed in the Vicon reference frame, the 3D location of the gaze is calculated from the intersection of the gaze vector with a horizontal plane, coinciding with the helicopter's current height.

### 6.5. Extracting and classification of gaze patterns

Basic eye movements include saccades, fixations, and smooth pursuit. *Saccades* are fast eye movements (about 200–600 °/s Boghen et al., 1974) that redirect the eye to a new location. Their duration ranges from 20 to 200 ms (reading typically involves saccades of 20–30 ms). There is usually a time delay between the appearance of a visual stimulus and the ensuing saccade, as well as a minimal time period between saccades. *Fixations* are the intervals when the gaze is stabilized on a typically stationary point of interest. The fixation is used to acquire relevant information about the environment. The duration of eye-fixations varies within a range of 150–600 ms. *Smooth pursuits* are eye-movements used to track moving visual stimuli. The pursuit motion can only occur in the presence of a moving target. Smooth pursuit employs a mechanism to stabilize the retina and coordinate with the body movement (mainly head movement). Micro saccades usually accompany smooth pursuit as a correction mechanism for eye position. It's during fixations and smooth pursuits that high-quality visual information is acquired. Gaze stability is required to prevent blur, due to the relatively large retinal photoreceptors' time constant (Land, 1999). Because of the high velocity of saccades, the visual system is blind during those eye motions.

**Figure 7:**
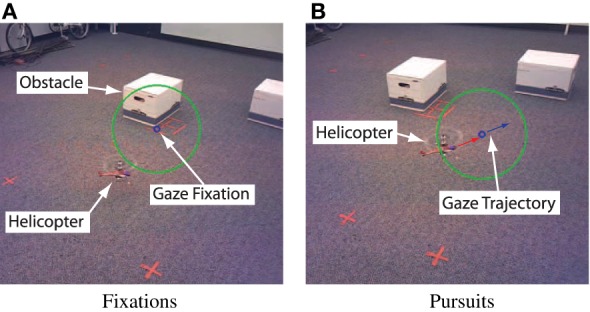
**Typical fixations and smooth pursuits, as seen from the ETG's camera**. The fixations tend to be discrete points driven by saccadic motions. During pursuits the gaze follows the helicopter, and its center remains within a 15° sector around the fovea (shown by the circle). This region represents the region of high acuity and seems to play an important role in visuo-motor control.

**Figure 8:**
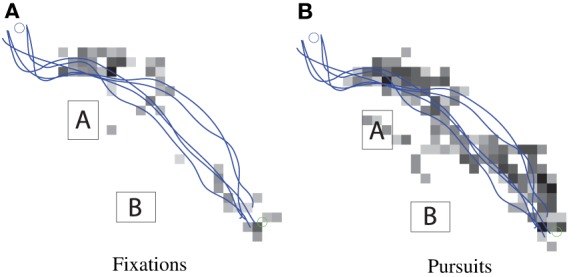
**Spatial density distributions of gaze fixation and pursuit in the task global reference frame for a sample of trajectories**. Dark cells represent areas favored by the gaze.

**Figure 9:**
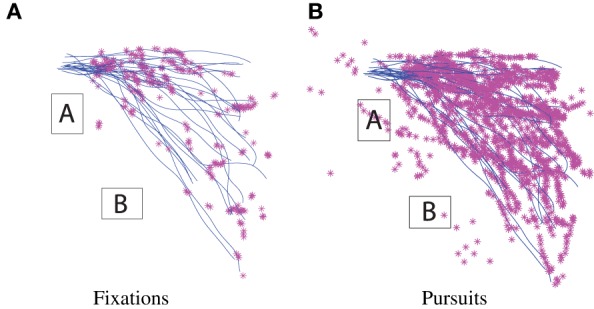
**Spatial distributions of gaze fixation and pursuit in the task global reference frame for the corresponding extracted IP**. The markers are gaze location samples, and the blue lines are trajectory segments associated with this particular IP.

These three basic eye-movements, saccades, fixations, and smooth pursuit, are classified using their specific kinematic characteristics. Figure 7 shows typical scenes from the ETG's cameras during pursuit and fixation. These scenes show the interaction between helicopter and gaze motion in the surrounding environment. In the following results, gaze classification was achieved by specifying the respective thresholds of gaze velocity and duration. Density plots for the gaze, classified into fixation and pursuit over a small trajectory sample, are shown in Figures 8A,B. Figures 9A,B show the fixation/pursuit distributions of a sample IP.

### 6.6. Results: fixation and pursuit gaze patterns

The fixation density plot (Figure 8A) shows that fixations are primarily concentrated around starting locations and the NE corner of obstacle A. This data is consistent with the definition of fixation. When superimposed with the helicopter's trajectories, it can be seen that these areas coincide with the subgoal regions associated with the IP.

The smooth pursuit gaze-density plot (Figure 8B) shows that gaze pursuit motion coincides with the helicopter trajectories. However, it's unclear whether the eye is tracking the helicopter to determine location and speed, or if it's engaging in visuo-motor control. Further investigation will be required to understand the dynamic relationship between the gaze and helicopter control (Andersh et al., 2014).

### 6.7. Results: agreement between gaze patterns and attentional model

Based on the hierarchic model, switching between IPs occurs at subgoals. If subgoals play a central role in the organization of behavior, anticipatory gaze patterns can be expected at these locations. The fixations in Figure 9A, extracted for a sample IP, show significant overlap between fixation points and subgoal region.

On the whole, the operator spends the majority of the time attending to the helicopter via smooth pursuit, as shown by the respective number of gaze samples in Figures 9A,B. Intermittent operator eye saccades are observed at locations ahead of the helicopter. These fixations coincide with the subgoal areas or obstacle corners. Furthermore, as expected from the attentional model, when the helicopter is approaching a subgoal, the gaze makes anticipatory saccades toward the subsequent subgoal region.

## 7. Discussion

This section returns to the topics that were introduced in Sections 2 and 3.5.

The proposed model describes how behavior is decomposed into discrete elements, i.e., the IPs, and therefore provides a link to the important concept of chunking and subgoals. The present work makes no assumption about the process generating subgoals. The IPs are extracted using a data-driven process. The paper demonstrates that (1) human operators, indeed, organize behavior following subgoals and (2) the use of equivalence is a fundamental principle driving this process. Existing strategies to determine subgoals, such as visibility graphs, are not necessarily feasible given the constraints of motion (vehicle) dynamics and do not account for the perceptual and other constraints affecting human process. The results demonstrate that subgoals emerge from the brain's need for efficient information processing. The brain achieves this goal by exploiting the similarities/invariants in the problem space.

The model addresses both the *system* and *behavioral complexity* brought up in the introduction. The IP encapsulates the similarities in strategies (equivalence classes) available in spatial behavior. Together with the symmetry group property associated with rigid body motion, they make it possible to use these IPs as units of behavior. Together, these properties enable systematic organization of the behavior with a large reduction in computational complexity. One way to appreciate the reduction in complexity is to consider the use of a DP with full state representation, comparing this program to a representation that uses IPs as states. Note the dramatic reduction in the size of the state space and hence, “curse of dimensionality.” In addition, the IPs result in a hierarchical representation, where the control modulation is performed largely independent of planning. This functional property directly addresses issues arising from the *system complexity*. This functional hierarchy also leads to reduction in attention load (information extraction). In the full state problem formulation, the operator has to attend to the complete state vector. With the IP, the operator needs to attend to the subgoal state and necessary information within the IP class, needed for modulation of the behavior.

Furthermore, it was shown that the IP can be easily abstracted using TTG functions. The TTG provide an efficient representation for planning. As discussed previously, human planning mechanisms may not follow wavefront algorithm, nevertheless, the fact that behavior within IPs is coherent and easily abstracted supports their role as a unit of behavior. This result extends the earlier results obtained by mapping behavior for simple goal interception tasks (Mettler and Kong, 2013).

The model also outlines how the behavior is “articulated.” The behavior's degrees of freedom, when described in terms of IP, are the subgoals, and to some extent the behavior within the IP. These subgoals could potentially be modified dynamically and the behavior within the IPs modulated to adapt behavior to small perturbations. Note that the mechanisms suggested by the properties of the model still need to be validated.

The model provides insights into how these mechanisms are implemented perceptually. First, the hierarchic model allows a clear delineation of the perceptual functions and the attentional elements associated with each level of the hierarchy. The gaze-tracking measurements taken during the guidance experiments show it's possible to classify eye motions according to smooth pursuits, saccades, and fixations. The preliminary gaze pattern analysis reveals that gaze behavior is consistent with the patterns suggested by the hierarchic attentional model. With this additional link between attention and behavior, the concept of IP could be regarded as a coordinated ensemble of equivalent control behaviors, with their associated perceptual behaviors and cues. For example, perceptual guidance could then be viewed as an instance of IP.

Finally, the results obtained are generally dependent on the subject's skill level. The experiments used here were conducted with operators of intermediate skill. In previous and ongoing investigations, it was found that the general model is valid. As skill levels increase, there is a better definition of the IPs and a better-differentiated dynamic mode (see Kong and Mettler, 2013). Similar trends should be expected for gaze patterns. For more details on the effects of skill see Li and Mettler (2013). The model is also currently being extended for skill analysis in surgery Li et al. (2014).

## 8. Conclusion

The paper presented a hierarchic model of human guidance, building on the concept of IPs, which are defined based on invariants inherent to guidance behavior. The IPs provide the natural building blocks needed for modeling the hierarchical organization of behavior, making it possible to delineate the structural and functional levels associated with control, guidance, and planning. The model delineates the physical and low-level control associated with vehicle guidance and control and the higher-level organization of guidance behavior, in terms of the IPs and their associated subgoals.

These results also underscore that investigating the entire system hierarchy and interactions is key to understanding the various mental and sensory processes allowing humans to achieve their agile and versatile guidance and control performance. More generally, the model addresses the system and behavioral complexity. Moreover, it does this without introducing undesired constraints. This is essential, given that the research goal is to understand principles and mechanisms underpinning versatility and adaptability.

Overall, the results show that the model provides a systematic and physically grounded framework that will make it possible to integrate attention, response modulation, and adaptation. The benefit of this model is that it is verifiable, i.e., every element of the model can be systematically tested. These aspects will be further investigated using experiments that incorporate dynamic and uncertain task elements, which will then be applied to other domains of human control and guidance skills. The future investigation will also focus on analyzing guidance behavior and gaze patterns together in dynamic and uncertain conditions.

The model is also relevant to a broad range of scientific and engineering applications. The model's explicit description of the primary control, planning, and perceptual functions will help build cognitive models, which are necessary for studying human factors in spatial control task. In particular, the effects of such issues as limitation of working memory and attention capacity on behavior are critical for various forms of transportation. The coupling with attention will assist researchers in achieving a more detailed understanding of effects of attention limitation and workload concepts. It will also help illuminate skill acquisition, while improving techniques based on learning from human demonstration. Finally, the model and gained understanding will aid the design of human-machine systems and the development of novel guidance algorithms for autonomous vehicles.

### Conflict of interest statement

The authors declare that the research was conducted in the absence of any commercial or financial relationships that could be construed as a potential conflict of interest.

## Footnotes

^1^Note that this example is representative of a range of spatial control problems that combine the agent dynamics, an environment, and some goals. These elements can be substituted for those corresponding to another problem domain
